# The underlying mechanism of prodromal PD: insights from the parasympathetic nervous system and the olfactory system

**DOI:** 10.1186/s40035-017-0074-8

**Published:** 2017-02-20

**Authors:** Shu-Ying Liu, Piu Chan, A. Jon Stoessl

**Affiliations:** 1 Department of Neurobiology, Neurology and Geriatrics, Xuanwu Hospital Capital Medical University, Beijing, 100051 China; 2 Beijing Key Laboratory on Parkinson’s Disease, Parkinson Disease Center of Beijing Institute for Brain Disorders, Beijing, 100051 China; 3 Pacific Parkinson’s Research Centre, Division of Neurology and Djavad Mowafaghian Centre for Brain Health, University of British Columbia and Vancouver Coastal Health, Vancouver, V6T 1Z3 BC Canada

**Keywords:** Parkinson’s disease, Prodromal, Alpha-synuclein, Parasympathetic nervous system, Olfactory system, Subtype

## Abstract

Neurodegeneration of Parkinson’s disease (PD) starts in an insidious manner, 30–50% of dopaminergic neurons have been lost in the substantia nigra before clinical diagnosis. Prodromal stage of the disease, during which the disease pathology has started but is insufficient to result in clinical manifestations, offers a valuable window for disease-modifying therapies. The most focused underlying mechanisms linking the pathological pattern and clinical characteristics of prodromal PD are the prion hypothesis of alpha-synuclein and the selective vulnerability of neurons. In this review, we consider the two potential portals, the vagus nerve and the olfactory bulb, through which abnormal alpha-synuclein can access the brain. We review the clinical, pathological and neuroimaging evidence of the parasympathetic nervous system and the olfactory system in the neurodegenerative process and using the two systems as models to discuss the internal homogeneity and heterogeneity of the prodromal stage of PD, including both the clustering and subtyping of symptoms and signs. Finally, we offer some suggestions on future directions for imaging studies in prodromal Parkinson’s disease.

## Background

Parkinson disease (PD), characterized by its motor symptoms (bradykinesia, resting tremor, and rigidity) [1], does not start suddenly. By the time the clinical diagnosis has been made, some 30–50% of dopaminergic neurons have been lost in the substantia nigra [2]. Symptomatic treatments are effective in most patients with PD, but currently no drugs have demonstrated convincing evidence of disease modification. One possible explanation is that the pathology of PD may be sufficiently advanced at the point of diagnosis that none of the interventions can rescue the remaining dying neurons, thus the prodromal stage of PD, during which the disease pathology has started but is insufficient to result in clinical manifestations, provides a valuable window during which disease-modifying therapies can be tested [3].

According to recent Movement Disorder Society criteria, early PD can be divided into three stages: preclinical PD (neurodegeneration has started yet without evident symptoms and signs); prodromal PD (symptoms and signs are present, but are still insufficient to define PD) and clinical PD (diagnosis of PD based on classical symptoms). The criteria are based upon probability and likelihood since it is not possible to identify prodromal PD with 100% certainty; probable prodromal PD is defined as a high likelihood (greater than 80%) and possible prodromal PD as a likelihood between 30 and 80% [4, 5]. The cardinal features of prodromal PD are non-motor and include constipation, hyposmia/anosmia, depression, REM sleep behavior disorder, orthostatic hypotension, and loss of heart rate variability [6]. Notably, many of the symptoms that emerge earlier in the disease course can be attributed to dysfunction in the peripheral nervous system or the peripheral part of the central nervous system, such as the vagus nerve (e.g. constipation), the sympathetic nervous system (e.g. orthostatic hypotension), or the olfactory bulb (hyposmia).

Neuronal aggregation of alpha-synuclein (α-syn) in Lewy bodies and Lewy neurites, the pathological signature of sporadic PD, can be found in the peripheral nervous system of PD patients [7]. It is not clear whether these structures are the original site of α-syn aggregation or whether they are subject to α-syn pathology transported from the brain. In support of the former hypothesis, truncal vagotomy has been associated with a reduced risk of PD after 20 years of follow-up (adjusted hazard ratio [HR] = 0.53; 95% CI: 0.28–0.99) [8]. Based on evidence from human studies, cell culture and animal models, the paradigm of pathological protein propagation in neurodegenerative diseases has been extended to include the concept that pathology arising from neurodegeneration-related proteins such as α-syn, amyloid-β, tau and TAR DNA-binding protein 43 (TDP43) may propagate in a prion-like fashion [9–13]. On the other hand, the prion hypothesis as selective neuronal vulnerability may be another important factor contributing to specific patterns of degeneration in human and animal brains [13]. In PD patients who underwent human fetal nigral transplantation, Lewy body-like inclusions that stained positive for α-syn were found in the grafted nigral neurons 14 years after transplantation, suggestive of cell to cell transmission [14, 15]. It is hypothesized that the propagation of α-syn in the brain starts in the dorsal motor nucleus of the glossopharyngeal and vagus nerves (DMV) and the olfactory bulb; from these two structures the α-syn pathology spreads in an ascending pattern to the pons, the midbrain, the basal forebrain and finally to the neocortex through chains of vulnerable neurons [16–18]. The so-called “Braak hypothesis” provides a mechanistic underpinning for the prodromal stage of PD, as non-motor symptoms could be explained by pathology in the peripheral nervous system and caudal brainstem that precede the onset of classic motor symptoms which do not emerge until Lewy pathology affects the substantia nigra. In this review we consider the two potential portals through which abnormal α-syn can access the brain: the vagus nerve and the olfactory bulb. We review clinical, pathological and neuroimaging evidence, and suggest future directions for studies in prodromal disease.

## Constipation and the parasympathetic nervous system

### Risks of PD

Constipation is a non-specific yet sensitive prodromal symptom of PD (sensitivity 79%, specificity 31% from Honolulu-Asia Aging Study) [19, 20]. At 10 years before diagnosis of PD, the incidence of constipation was already higher in those who went on to develop PD than in controls (relative risk [RR] = 2.01; 95% CI: 1.62–2.49) while the incidence of other typical prodromal symptoms (except tremor) fails to reach significance until 5 years before diagnosis [21]. To date, eight large longitudinal cohorts confirmed the increased risk of PD in populations with chronic constipation [19, 21–27], providing sufficient evidence for the Movement Disorder Society task force to calculate a likelihood ratio (LR) for constipation in the research criteria for prodromal PD (constipation LR + = 2.2, LR− = 0.8) [5].

### Underlying mechanisms and the role of α-syn

The mechanism of constipation in PD and prodromal PD is still under debate. A-syn deposition and Lewy type α-syn pathology affecting the gastrointestinal tract have been frequently reported from biopsy and postmortem studies; however, the types of antibodies, the morphological assessment of pathology and the site of biopsy varied considerably, in line with the inconsistent measures of sensitivity and specify of α-syn pathology detected between patients and healthy aged controls [28, 29]. Among the many contradictory results, one of the more consistent findings is a rostral-caudal gradient of α-syn pathology throughout the gastrointestinal canal (most dense in the lower esophagus, stomach, and upper small intestine; lowest in the colon and rectum) [7, 30], which correspond to the rostral-caudal gradient of vagal innervation [31]. The DMV is one of the earliest sites of α-syn aggregation in the central nervous system according to Braak, and more than 50% of efferent motor neurons were already lost by the time that clinical PD became manifest [32]. It is hypothesized that the accumulation of α-syn may originate in the enteric nervous system and be transported in a retrograde manner through the vagus nerve. By inducing normal α-syn to misfold in a prion-like manner, the cycle may repeat itself and lead to self-propagation and cell loss in networks of connected neurons [13].

In retrospective pathological studies of PD patients who underwent colon biopsy years before being diagnosed with PD, α-syn pathology in the gastrointestinal tract could be detected up to 20 years prior to the full manifestation of PD symptoms [33–35]. In one study of patients with REM sleep behavior disorder (RBD), which carries a high risk of future synucleinopathy, immunostaining of phosphorylated α-syn was reported in four of 17 subjects, whereas none of the 14 healthy controls was positive [36]. Even though these findings support the accumulation of α-syn in the gut as a possible peripheral mechanism for constipation, caution is required owing to inconsistency of findings and the absence of direct evidence of centripetal spread of α-syn in humans.

There is recent evidence for alterations in the gut microbiome in PD [37–39]. Whether gut microbial content is altered as a manifestation of impaired colonic motility or whether altered GI flora can result in regional neurotoxicity remains to be determined.

### Evidence from medical interventions

Based on clinical and pathological evidence, further investigations were conducted into the potential neuroprotective effects of gastrointestinal interventions such as vagotomy and appendectomy. A small cohort with 34 patients who underwent appendectomy before PD onset showed that past appendectomy may be associated with more years of life without PD symptoms (*P* = 0.040) [40], however, a later population-based study of 265,758 patients with appendectomy and 1,328,790 comparison controls indicated no difference in risk of PD between subjects with or without appendectomy in mid or late life (HR = 1.00; 95% CI: 0.74–1.36) [41].. On the other hand, Svensson et al. assembled a population-based registry-linkage cohort with 14,883 patients who underwent vagotomy between 1977 and 1995 and analyzed the incidence rates and HR of PD afterwards, the overall adjusted HR between patients with truncal vagotomy was 0.85, 95% CI: 0.63–1.14; for those with follow-up of more than 20 years, adjusted HR was 0.53, 95% CI: 0.28–0.99 [8]. The study is the first evidence that by preventing vagal transport, the risk of PD decreased, supporting a possibly critical involvement of the vagus nerve in the pathogenesis of PD.

### Evidence from imaging

Positron emission tomography (PET) offers a useful tool to investigate physiological dysfunction in vivo [42]. In 2014, the PET tracer 5-^11^C-methoxydonepezil was validated for the in vivo quantification of acetylcholinesterase (AChE) density in humans and thus can serve as a biomarker for parasympathetic dysfunction. Significantly decreased ^11^C-donepezil standard uptake values in the small intestine and pancreas were detected in twelve PD patients compared to age-matched controls (small intestine: −35%, *P* = 0.003; pancreas: −22%, *P* = 0.001); the results were similar when distribution volume was assessed (small intestine: PD 66.4 ± 15.4 control 111.9 ± 40.0, *P* = 0.001; pancreas: PD 126.2 ± 31.7 control 167 ± 64.2, *P* = 0.061) [43]. Interestingly, the rostral-caudal pattern of vagal innervation was replicated by the distribution of ^11^C-donepezil binding: highest in the upper gastro-intestinal tract and lower in the ileum and colon. This study supports suggestions of impaired vagal activity in PD patients but there was no relationship between reduced cholinergic activity and severity of PD. However, reduced ^11^C-donepezil uptake is not specific for decreased vagal innervation, as it might also reflect the loss of cholinergic enteric neurons.

## Hyposmia and the olfactory system

### Risk of PD

The other potential portal for aggregated α-syn to enter the central nervous system are the anterior olfactory structures. Olfactory loss demonstrated by objective test is the only non-motor symptom that has more than 80% specificity for the differential diagnosis of PD from other parkinsonian conditions in the MDS clinical diagnostic criteria [1]. Hyposmia is also predictive of the future development of clinical PD in both general and high-risk populations, but with lower specificity (sensitivity 79%, specificity 53% from Honolulu-Asia Aging Study; sensitivity 60%, specificity 72.6% from Prospective Validation of Risk factors for the development of Parkinson Syndromes study) [20, 44, 45]. Based on the predictive value of olfactory dysfunction and dopaminergic deficit in dopamine transporter (DAT) imaging, the nested population-based Parkinson Associated Risk Syndrome study was launched from 2008: 4999 subjects completed a 40-item University of Pennsylvania Smell Identification Test (UPSIT) in the first stage; 203 hyposmic subjects and 100 normosmic subjects underwent ^123^I-ß-CIT/SPECT at the baseline of the second stage [22, 46]. The results demonstrated a significant predictive ability of hyposmia for dopaminergic dysfunction (odds ratio [OR] = 12.4, 95% CI: 1.6–96.1) at baseline and a 61% phenoconversion rate of subjects who had both hyposmia and DAT deficit (of whom there were only 23) in the 4-year follow-up [47]. For high-risk populations, Postuma et al. reported that the UPSIT scores of RBD patients who developed PD in 10 years were much lower at baseline than RBD patients who remained disease-free (HR = 2.8, 95% CI: 1.3–6.0, *P* = 0.003) [48]. Similar results were found in an RBD cohort from Spain and in a cohort of first degree relatives of PD [49–51]. The Movement Disorder Society task force determined a LR+ of 4.0 and a LR− of 0.43 for olfactory dysfunction in the research criteria for prodromal PD [5].

### Underlying mechanisms and the role of α-syn

Hyposmia/anosmia in PD could reflect both cortical and local pathological changes and likely involves a complex integration of central network deficits and local neural dysfunction, in which the role of α-syn may be critical. The olfactory receptor neurons are directly exposed to the external environment and thus prone to attack from viruses, toxins or other pathological particles. The axons of the olfactory neurons pass though the cribriform plate and reach the mitral or tufted cells in the olfactory bulb, whose axons project in turn to the anterior olfactory nucleus, the piriform cortex, the periamygdaloid cortex, the olfactory amygdala and entorhinal cortex [52, 53]. A-syn pathology in the olfactory mucosa of PD patients does not appear to be greater than that in healthy age-matched controls [54, 55], while in the olfactory bulb there is evidence for abnormal α-syn deposition that distinguishes PD subjects from healthy elderly controls with a sensitivity of 95% and a specificity of 91% [56]. The anterior olfactory nucleus, which receives input from the mitral and tufted cells, was the most heavily involved structure in the bulb region; the cortical nucleus of the amygdala, which receives input from the primary olfactory bulb projections, exhibited considerably more α-syn pathology and neuronal loss than other amygdaloid nuclei [53, 56]. The extent of α-syn pathology in other brain regions, including substantia nigra, amygdala, cingulate cortex and orbitofrontal cortex, was strongly correlated with pathological burden in the olfactory bulb in the brains of patients with Lewy body diseases [56, 57]. In a small cohort of PD and incidental Lewy body disease cases, α-syn pathology was found in all sub-regions of the primary olfactory cortex. Despite the fact that all the sub-regions are separated from the olfactory bulb by only a single synapse, the burden of α-syn pathology varies: highest in the frontal and temporal piriform cortex and lowest in part of anterior entorhinal cortex [58]. Together, these results support the possibility that the pathology of PD spreads along olfactory pathways but is additionally influenced by differential neural vulnerability.

Evidence from animal models showed that after injection of preformed fibrils of recombinant α-syn into the olfactory bulb, wild-type mice developed not only olfactory deficits, but also α-syn pathology in brain areas unconnected to the olfactory system after a time interval of about half a year [59]. Similar changes were seen following intranasal instillation of pro-inflammatory lipopolysaccharide [60]. Widespread propagation of α-syn pathology through connected anatomical pathways was observed in the animal study: 1 month after intranasal injection, α-syn phosphorylated on serine 129 (Pser129) was found in areas directly connected to the olfactory bulb, including piriform cortex, entorhinal cortex and cortical amygdaloid nuclei; 3 months after, the pathology had progressed to those brain areas one synapse removed from the olfactory bulb, including the hippocampus, insular cortex and frontal cortex; by 6 months Pser129-positive cells were found two synapses removed from the olfactory bulb and 12 months later Pser129 pathology was widespread in cortical associative and secondary cortical brain regions, somatosensory cortex and the anterior cingulate area [59]. The propagation model was created using preformed fibrillary assemblies of recombinant α-syn in mice, thus may provide only an indirect simulation of the behavior of α-syn in the human olfactory system.

In the aged human population, a postmortem study was performed in 164 participants who underwent olfactory testing during the longitudinal Honolulu-Asia Aging Study; incidental Lewy bodies were found in the substantia nigra or locus coeruleus in only 1.7% of subjects in the highest tertile of olfactory performance, but in 18.2% of subjects in the lowest tertile, with an age-adjusted OR of 11.0 (95% CI: 1.3–526) [61]. In another study with 320 consecutive autopsies from a general geriatric hospital, α-syn pathology restricted to the olfactory bulb was detected in 16 subjects (2% of all participants), of whom two had α-syn pathology in the anterior olfactory nucleus alone, and 14 in the peripheral olfactory bulb [62]. In accordance with the results from previous studies, the extent of α-syn pathology in the amygdala was strongly correlated with that in the olfactory bulb (Spearman correlation R [R_S_] = 0.853) [56, 62]. Similar results were reported from elderly subjects with incidental Lewy body disease or Alzheimer’s disease with Lewy bodies [7, 63].

### Evidence from imaging

#### Anterior olfactory structures

Morphological analysis by structural magnetic resonance imaging (MRI) can be used to provide quantitative measurements of anatomical changes of brain structures, including volume, cortical thickness or shape. A meta-analysis of six case-control studies showed significant reduction of olfactory bulb volume in PD patients compared to heathy controls, the pooled weighted mean difference was −8.07 mm^3^ (95% CI: −14.72, −1.42) for the right olfactory bulb and −10.12 mm^3^ (95% CI: −16.48, −3.77) for the left olfactory bulb [64]. However, the results must be interpreted with caution as the heterogeneity between studies was quite high (I^2^ = 76%). Another study compared the volume of both olfactory bulb and tracts between patients with PD and with other forms of parkinsonism including progressive supranuclear palsy (PSP), multiple system atrophy (MSA), and corticobasal degeneration (CBD) and detected the lowest volume of 198.3 ± 60.1 mm^3^ in patients with PD, followed by 261.7 ± 75.5 mm^3^ in PSP, 278.2 ± 77.0 mm^3^ in MSA, 312.4 ± 30.2 mm^3^ in CBD, and 314.6 ± 42.6 mm^3^ in controls [65]. Using diffusion tensor imaging (DTI), two studies reported a significant increase of mean diffusivity, presumed to reflect axonal and myelin damage, in bilateral olfactory tracts of the PD patients. The mean diffusivity values of the olfactory tract and substantia nigra were significantly correlated with decreased 6-[^18^F]-fluorolevodopa uptake in the putamen (*R* = −0.71, *P* < 0.01; *R* = −0.52, *P* < 0.05 respectively) [66, 67]. The findings implied that microstructural degradation of the olfactory tract and the substantia nigra parallels progression of putaminal dopaminergic dysfunction, but the time sequence of the pathological changes cannot be determined from these studies. MRI and DTI measurements of olfactory bulb/tract degradation were associated with decreased olfactory performance [68, 69].

### Network and neural transmitter systems

The process of odor identification requires short-term working memory to receive test information and long-term memory to recognize and name the odor, so a normal olfactory performance requires the integrity of both primary olfactory cortex and higher order cognitive network such as the limbic network and is modulated by varies neural transmitters [70].

Focal voxel-based morphology analysis of the olfactory sulcus showed smaller depth in the PD patients but this did not correlate with olfactory identification performance [68], while the grey matter volume in the piriform cortex was positively correlated with the olfactory performance in early PD subjects [71].

In both PD and healthy controls, olfactory stimulation activated vast brain regions in functional magnetic resonance imaging, including amygdaloid complex, hippocampal formation, lateral orbitofrontal cortex, striatum, thalamus and midbrain; compared to control subjects, the activation in amygdala and hippocampal formation was reduced in PD patients [72]. In a study using olfactory event-related potentials to identify hyposmia, further decrease of activation was found in the inferior frontal gyrus, insula and cingulate cortex as well as in amygdala and hippocampus in PD without identifiable olfactory event-related potentials [73]. Other cortical regions with decreased activation in hyposmic PD included medial frontal gyrus, middle temporal gyrus and occipital cortex [74]. In resting state, the regional homogeneity and functional connectivity within primary olfactory cortices and secondary olfactory structures were reduced in hyposmic PD; along with significantly decreased connectivity within limbic/paralimbic networks between gyrus rectus and orbital frontal cortex, parahippocampal gyrus, middle occipital gyrus, insula, temporal pole, posterior cingulate and amygdala [75]. A longitudinal ^18^F-fluorodeoxyglucose PET study showed reduced metabolism in bilateral medial prefrontal cortex and parieto-occipito-temporal cortex in hyposmic PD at baseline and a marked metabolic reduction in the posterior regions such as posterior cingulate, precuneus, medial occipital and parieto-occipito-temporal cortex at 3-year follow-up; this pattern of reduced metabolism has some extent of similarity with the PD-related cognitive pattern reported by the Eidelberg group [76, 77]. The PD group with hyposmia had significant deteriorations in Mini-Mental State Examination score compared to normosmic PD and one standard deviation change in the olfactory score at baseline resulted in 18.7-fold increase in the risk of developing PD with dementia in 3 years [76].

The connection between olfactory impairment and cognitive decline was further revealed by PET studies: positive correlations between UPSIT scores and acetylcholinesterase (AChE) activities were found in the hippocampal formation, amygdala and neocortex (*R* = 0.56, *P* < 0.0001; *R* = 0.50, *P* < 0.0001; *R* = 0.46, *P* = 0.0003; respectively); while limbic AChE activity also correlated positively with executive cognitive ability (*r* = 0.36, *P* = 0.006) and verbal memory (*r* = 0.29, *P* = 0.03) [70]. In the same study, higher UPSIT scores were associated with better scores on cognitive measures, revealing the same underlying cholinergic mechanism behind olfactory deficits and cognitive decline. To date, the linkage between hyposmia and cognitive disorder were reported from symptomatic level, structure level, resting-state and event-related functional level, metabolic level and neurotransmitter level [45, 70, 75, 76].

Olfactory function has been reported to correlate with the integrity of other neurotransmitter systems in PD, such as binding potential of vesicular monoamine transporter type 2 in the striatum (*R* = 0.30, *P* < 0.05) and binding potential of DAT in the hippocampus, amygdala and striatum (R_S_ = 0.54, *P* = 0.003; R_S_ = 0.43, *P* = 0.02; R_S_ = 0.48, *P* = 0.008; respectively) [70, 78]. There is lack of significant correlation between binding potential of serotonin transporter in the raphe nucleus, amygdala, hippocampus, striatum or neocortex [79], which is contradictory to the results from animals [80, 81]. A summary of important imaging evidence regarding parasympathetic nervous system and olfactory system was provided in Table 1. Association with decrease of odor identification capability and striatum DAT binding were also reported in general aged populations, patients with “idiopathic” olfactory loss and high-risk populations such as leucine-rich repeat kinase 2 (LRRK2) G2019S carriers [22, 44, 49, 82, 83]. However, it is difficult to know whether this reflects a true relationship between the dopaminergic loss and olfactory dysfunction or whether both findings might simply reflect underlying prodromal PD.

**Table 1:** Summary of pathological and imaging evidence of parasympathetic nervous system and olfactory system involvement in PD

Structure	α-syn pathology	Structural imaging	Functional imaging	Molecular imaging
Vagus nerve	Positive	NA	NA	NA
Gastrointestinal tract	Controversy	NA	NA	Decreased ^11^C-donepezil standard uptake values in the small intestine and pancreas following a rostral-caudal gradient [43]
Olfactory bulb	Positive	Bilateral reduction of olfactory bulb volume [64, 65, 68]	NA	NA
Olfactory tract	Positive	Bilateral increase of mean diffusivity [66, 67]	NA	NA
Olfactory cortex	Positive	Decrease of olfactory sulcus depth; decrease of piriform cortex volume [68, 71]	Reduced activation in amygdala and hippocampal formation after olfactory stimulation [72–74]; decreased regional homogeneity and functional connectivity within olfactory cortex and decreased connectivity within limbic/paralimbic networks [75]	Reduced glucose metabolism in bilateral medial prefrontal cortex and parieto-occipito-temporal cortex [76]; positive correlations between UPSIT scores and acetylcholinesterase activities in hippocampal formation, amygdala and neocortex [70]; positive correlations between UPSIT scores and vesicular monoamine transporter type 2 binding potential in striatum [70]; positive correlations between UPSIT scores and dopamine transporter binding potential in hippocampus, amygdala and striatum [78]

## The internal homogeneity and heterogeneity of prodromal mechanisms

In fact, the linkage between different prodromal symptoms and imaging signs of prodromal PD are universal. Hyposmia has been associated with constipation, depression, anxiety and mild motor symptoms [45], a combination of symptoms is more predictive of decreased DAT binding [22]. Other studies showed linkage between hyposmia, symptoms of autonomic failure and imaging evidence of sympathetic system denervation, such as lower cardiac septal: hepatic ratios of 6-^18^F-fluorodopamine-derived radioactivity and lower cardiac ^123^I-metaiodobenzylguanidine uptake [84–86]. In both manifest PD with RBD and idiopathic RBD patients, RBD has been linked with hyposmia, constipation, orthostatic symptoms, hallucinations, depression and worse parkinsonian sign [87, 88]. In population-based studies, substantia nigra hyperechogenicity has been associated with constipation, hyposmia, depression and mild parkinsonian signs [89].

The cause of this clustering of motor and non-motor symptoms is unknown, although different classifications of empirical subtypes based on the clusters are proposed [90], the phenomena may simply follow the severity of pathological development of PD. Hyposmia, RBD and constipation constantly appear in different clusters, while the corresponding pathological structures are either the potential portals for α-syn aggregation (DMV and olfactory system) or are close to them (locus coeruleus/subcoeruleus complex and pedunculopotine nucleus), so it is natural that the symptoms should cluster together if α-syn propagates though the relevant structures. In support of this view, some evidence showed possible higher α-syn burden in subjects with hyposmia, RBD and reduced ^123^I-metaiodobenzylguanidine uptake [91–93], in agreement with the Braak stage and the progression of PD. From this perspective, the homogeneity in the development of parkinsonian pathology is emphasized, and the recently described research criteria for prodromal PD assign each symptom and sign in those clusters into a combined score to predict future PD manifestation [5].

On the other hand, such a scheme may neglect important heterogeneity of mechanisms in the development of PD. Braak and colleagues have proposed a dual-hit hypothesis in which a neurotropic pathogen might enter the brain through either the gastrointestinal or the nasal route [94], either of which can result in disease progression, but potentially with different manifestations [95, 96]. Empirical nonmotor subtypes are recently proposed, which categorize patients into brainstem phenotype (brainstem route, characterized with late onset hyposmia, RBD and dysautonomia), limbic phenotype (olfactory route, characterized by anosmia, depression, fatigue and central pain) and cognitive phenotype (diffused, characterized by cognitive decline) [97, 98]. So far, no pathological evidence is available to support such subtyping and the internal axonal linkage between the olfactory bulb, olfactory cortex and basal forebrain, hypothalamus, and brainstem may introduce ambiguity in the separation of the two hypothetical routes [99, 100]. However, functional and structural network analysis based on neuroimaging may help to investigate the real propagation patterns of α-syn pathology in the brain.

Another illustration of heterogeneity in PD is based on genetic subtypes, as there is evidence of pathophysiological differences related to certain gene mutations, such as increased inflammation in LRRK2 mutation carriers [101, 102]. The lack or lesser extent of α-syn deposition in some genetic forms of PD further emphasizes these differences [103]. Compared to RBD patients, LRRK2 carriers have significantly lower prevalence of olfactory loss, cognitive decline or sleep disturbance in the prodromal stage [104–108]. Neuroimaging studies are needed to consider the functional and structural network changes in the genetic subtypes and to evaluate the differences between the sporadic subtypes and genetic subtypes in both non-manifest and manifest stages.

Even though not emphasized in this review, the sympathetic nervous system may deserve more attention in attempting to understand mechanisms of prodromal PD, as there is evidences for pre-motor involvement of peripheral noradrenergic depletion [109], while the noradrenergic nucleus locus coeruleus may be affected prior to the substantia nigra in the prodromal stage. Related biomarker such as ^123^I-metaiodobenzylguanidine uptake and 3-methoxy-4-hydroxyphenylglycol can be potential early indicators for central neurodegeneration [110].

## Conclusions

The underlying mechanism of prodromal PD includes both homogeneous and heterogeneous aspects. A-syn may proliferate in a prion-like manner and selectively cause neurodegeneration, which possibly represents as the Braak stage in pathology and lead to clusters of prodromal symptoms and signs in clinic; while the gastrointestinal tract/vagus nerve and olfactory system can be two separate routes and models of pathological progression. Further efforts are needed using neuroimaging as a tool to investigate the network changes.

## Abbreviations

AChE: Acetylcholinesterase
CBD: Corticobasal degeneration
DAT: Dopamine transporter
DMV: Dorsal motor nucleus of the glossopharyngeal and vagus nerves
DTI: Diffusion tensor imaging
HR: Hazard ratio
LR: Likelihood ratio
LRRK2: Leucine-rich repeat kinase 2
MRI: Magnetic resonance imaging
MSA: Multiple system atrophy
OR: Odds ratio
PD: Parkinson’s disease
PET: Positron emission tomography
Pser129: Alpha-synuclein phosphorylated on serine 129
PSP: Progressive supranuclear palsy
RBD: REM sleep behavior disorder
RR: Relative risk
TDP43: TAR DNA-binding protein 43
UPSIT: University of Pennsylvania Smell Identification Test
α-syn: Alpha-synuclein
